# Identification of a nanobody specific to human pulmonary surfactant protein A

**DOI:** 10.1038/s41598-017-01456-2

**Published:** 2017-05-03

**Authors:** Xian He, Shan-Mei Wang, Zhao Fang Yin, Meng-Meng Zhao, Nan Li, Feng Yu, Liu-Sheng Wang, Yang Hu, Yu-Kui Du, Shan-Shan Du, Yan Li, Ya-Ru Wei, Shan-Shan Chen, Jian-Hua He, Dong Weng, Hui-Ping Li

**Affiliations:** 1 Department of Respiratory Medicine Suzhou University, School of Medicine, SuZhou, China; 2 Department of Respiratory Medicine, Shanghai Pulmonary Hospital Tongji University, School of Medicine, Shanghai, China; 3 Shanghai Institute of Applied Physics, Chinese Academy of Sciences, Shanghai, China; 4 Present Address: Department of Respiratory Medicine The Sixth People’s Hospital of Nantong, Suzhou University, School of Medicine, SuZhou, China

## Abstract

Nanobody (Nb) is a promising vector for targeted drug delivery. This study aims to identify an Nb that can specifically target the lung by binding human pulmonary surfactant protein A (SP-A). Human lung frozen tissue sections were used for 3 rounds of biospanning of our previously constructed Nb library for rat SP-A to establish a sub-library of Nb, which specifically bound human lung tissues. Phage-ELISA was performed to screen the sub-library to identify Nb4, which specifically bound human SP-A. The binding affinity Kd of Nb4 to recombinant human SP-A was 7.48 × 10^−7^ M. Nb4 (19 kDa) was stable at 30 °C–37 °C and pH 7.0–7.6 and specifically bound the SP-A in human lung tissue homogenates, human lung A549 cells, and human lung tissues, whereas didn’t react with human liver L-02 cells, kidney 293T cells, and human tissues from organs other than the lung. Nb4 accumulated in the lung of nude mice 5 minutes after a tail vein injection of Nb4 and was excreted 3 hours. Short-term exposure (one month) to Nb4 didn’t cause apparent liver and kidney toxicity in rats, whereas 3-month exposure resulted in mild liver and kidney injuries. Nb4 may be a promising vector to specifically deliver drugs to the lung.

## Introduction

Molecular-targeted agents have attracted the attention of pharmaceutical industry since 1980 because of their apparent advantages, including localized accumulation, controlled release, low toxicity, and high bioavailability. Antibody-conjugated active targeted drugs, which have a targeting mechanism that is mediated by a specific antibody-antigen interaction, are particularly investigated intensively^1^. *In vitro* cell line studies have shown that antibody-conjugated doxorubicin, such as anti-CD19 antibody-conjugated doxorubicin, appears to kill cancer cells more potently and specifically than free doxorubicin^2,3^. In our previous study, we developed an anti-SP-A polyclonal antibody-conjugated dexamethasone liposome, which showed a higher localized and specific accumulation in the lung and superior efficacy to attenuate lung injury than free dexamethasone in a rat model of bleomycin-induced lung injury^4^. However, the anti-SP-A polyclonal antibody-conjugated dexamethasone liposome has a high molecular weight, and thus penetrates the target tissue poorly and shows long hepatic and splenic retention and strong immunogenicity, suggesting that anti-SP-A polyclonal antibody may not be an ideal pulmonary targeting vector. In 1993, Hamers-Casterman *et al*. first isolated a naturally occurring heavy-chain antibody devoid of light chains from llama^5–7^, which has a nanoscale molecular weight (12–15 kDa) and thus is named as “nanobody” (Nb). Nb is actually not a conventional antibody; instead, it refers to the recombinant derivative of the variable domain of natural camelid antibodies. Since its discovery, the potential of Nb as an effective tool for diagnosis and drug delivery has been widely explored in several therapeutic areas, such as cancer and infectious diseases^6,8–10^. HRE-2-targeted Nb has been tested to diagnose and treat HER2-positive breast cancer^9^, and the anti-EGFR Nb 8B6 shows high specificity and selectivity on cells overexpressing EGFR and may be used for *in vivo* tumor imaging^10^.

The application of Nb in lung-targeting therapy has also been investigate. Currently, the majority of lung-targeting drugs are delivered through inhalation^11–13^, such as inhaled corticosteroid. Drugs administered by inhalation may be effective to treat respiratory tract disease, but may be ineffective for pulmonary diseases that involve alveoli and interstitium, such as interstitial lung disease^14,15^. Thus, Nb could be a promising solution to specifically deliver drugs to the lung. Here, the current study aims to identify an anti-SP-A Nb that is specific to human lung tissues.

## Results

### Biopanning, identification, and expansion of anti-human pulmonary SP-A Nb

**Figure 1:**
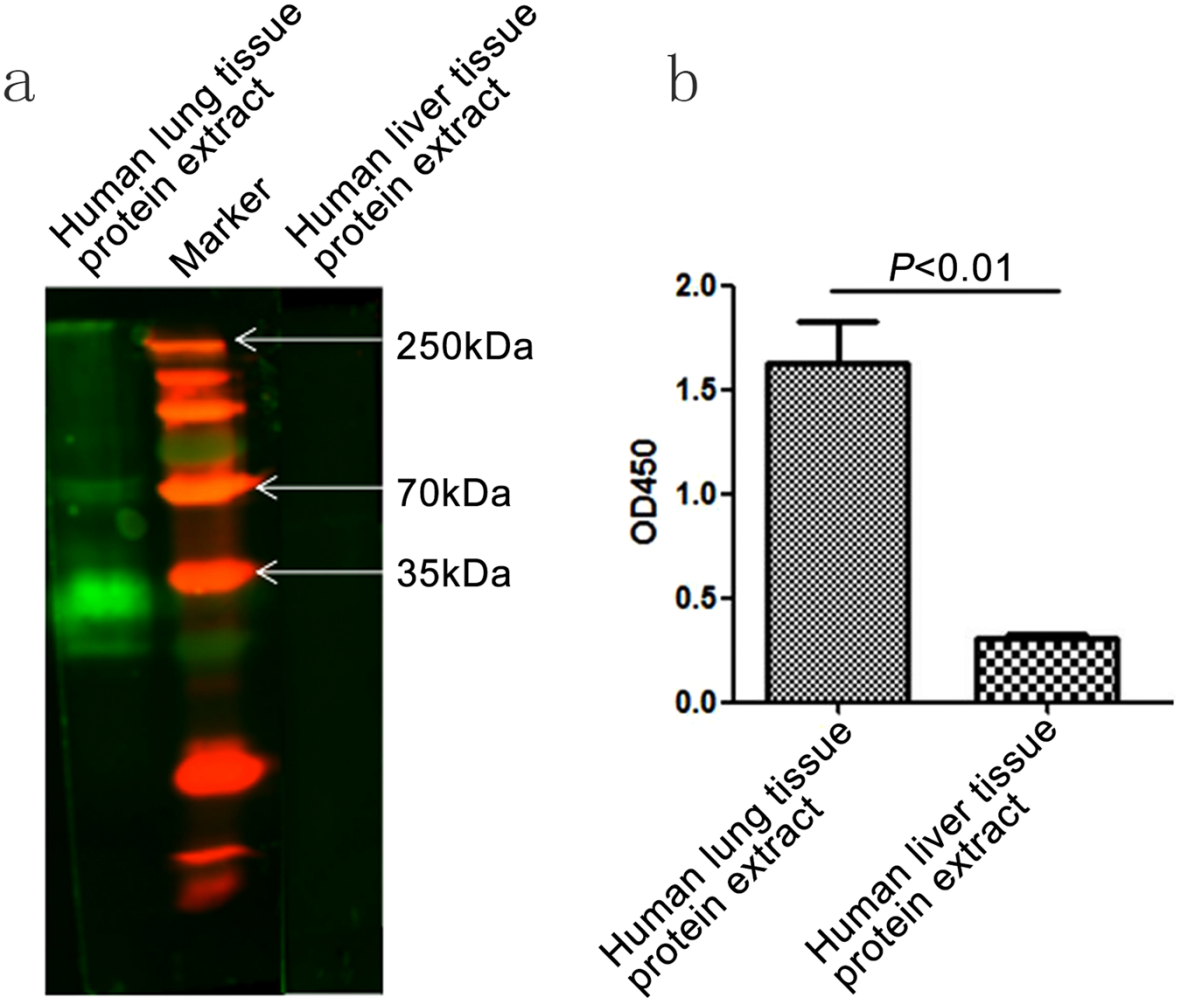
Human pulmonary tissues expressed abundant SP-A protein. (**a**) Representative image of Western blot for human pulmonary SP-A protein. Human pulmonary frozen tissues and human liver tissues were homogenized. Total proteins were extracted, separated (50 µg) on SDS-PAGE gel, and transferred to a PVDF membrane. The membrane was probed with the primary mouse anti-human SP-A monoclonal antibody (SP-A-mAb) and the secondary anti-mouse IgG-FITC antibody. (**b**) ELISA assay to detect human pulmonary SP-A protein. Human tissue protein extracts were added to 96-well plates, and the plates were incubated with SP-A-mAb and secondary antibodies. The absorbance at 450 nm (OD450) was determined in a microplate reader.

**Figure 2:**
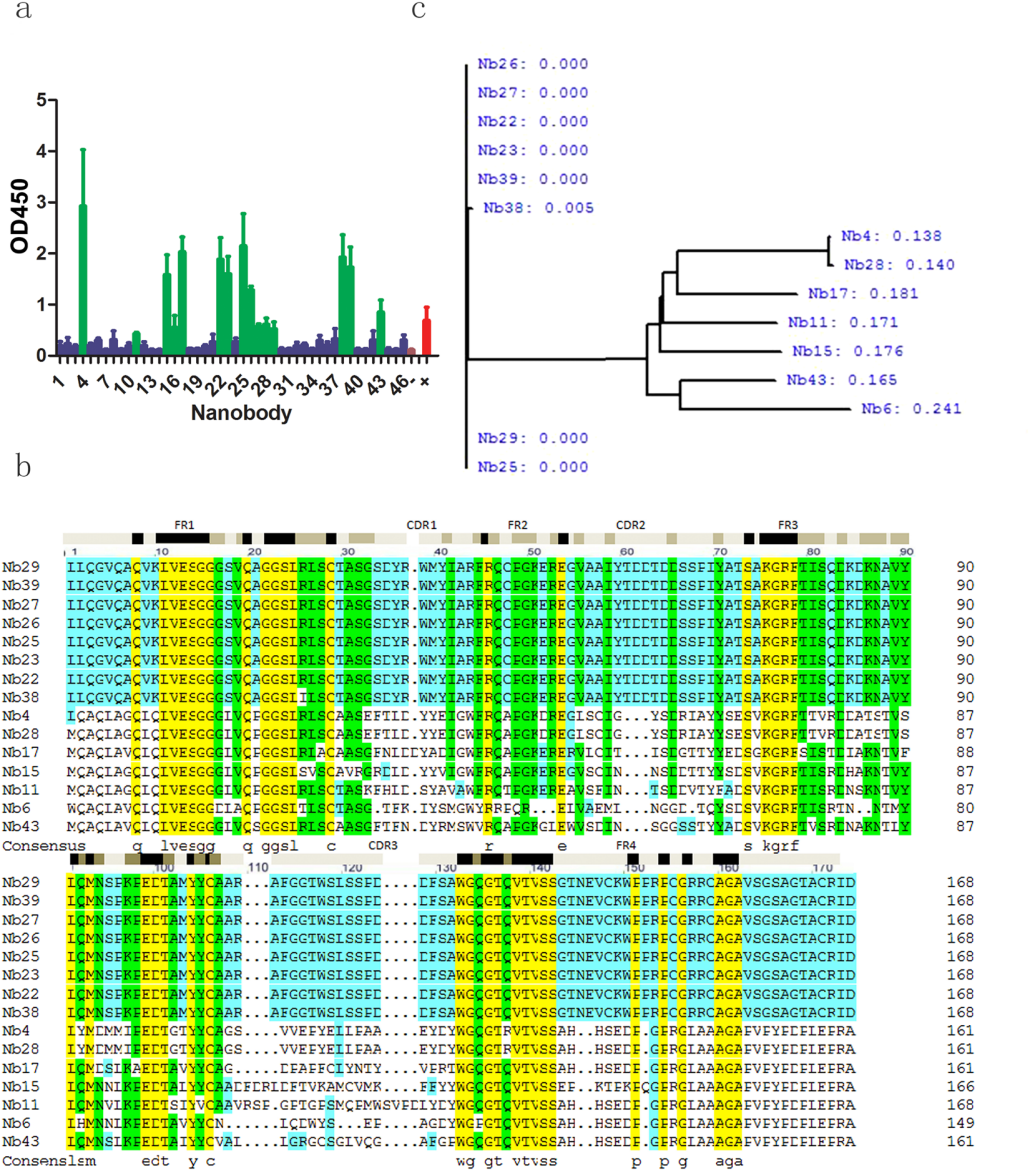
Identification of nanobody that specifically bound to human lung SP-A protein. (**a**) A total of 15 positive monoclonal Nbs were identified. (**b**) Peptide sequencing analysis revealed 4 IgG2a subtype clones and 11 IgG3 subtype clones. (**c**) Cluster analysis of the 15 peptide sequences found 7 different sequences and 2 overlapping sequences (Nb4 and Nb28). Phage-ELISA assay was performed using human pulmonary tissue total protein extracts as the antigen source and SP-A-mAb as the primary antibody.

**Figure 3:**
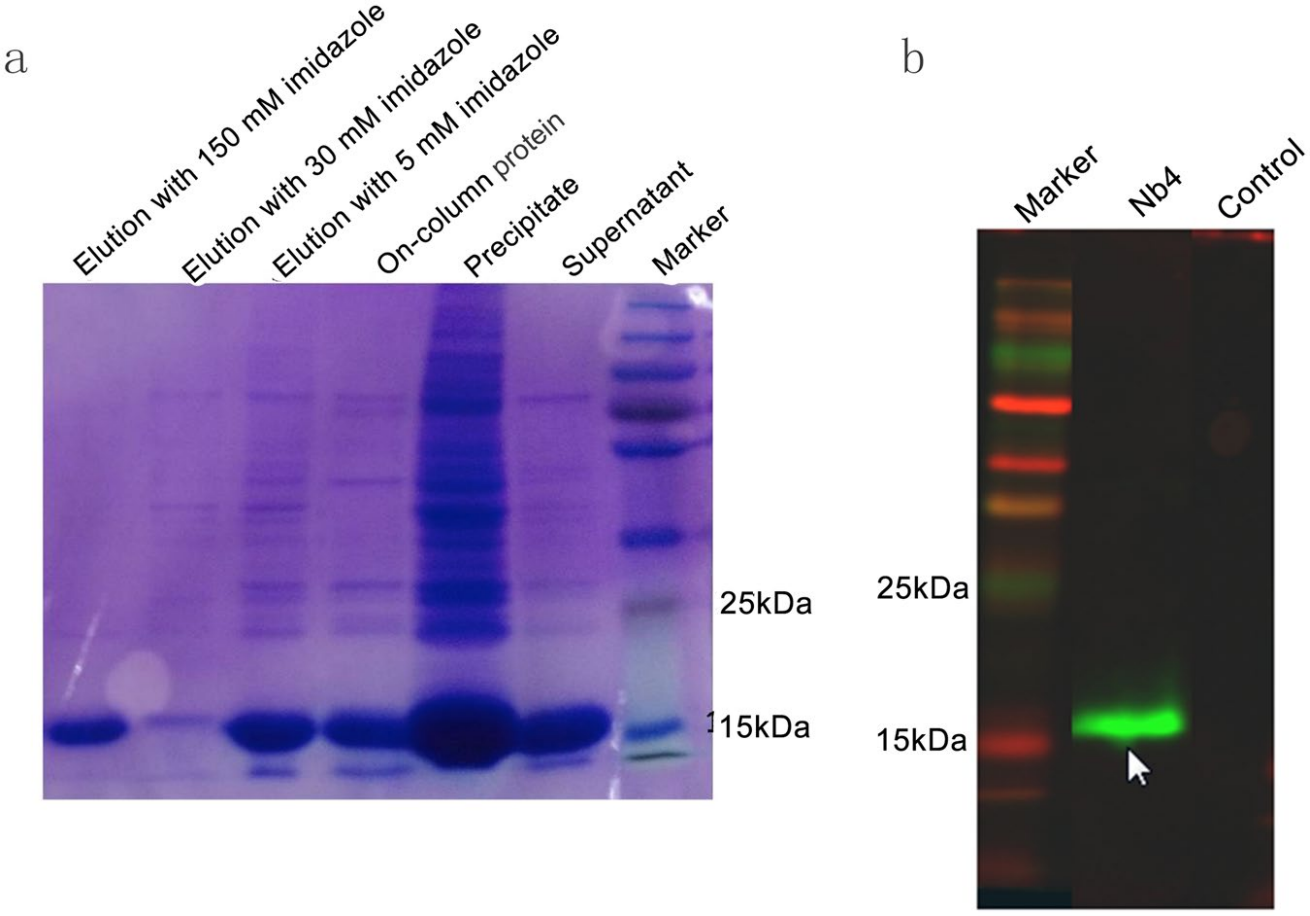
Expansion and characterization of Nb4. (**a**) Image of SDS-PAGE gel showing protein contents of different collections during Nb4 purification. The molecular weight of Nb4 is approximately 19 kDa. Nb4 was expanded by culturing bacteria that expressed Nb4 and purified. The supernatant, bacterial pellets, and different elution fractions were loaded on SDS-PAGE gel. (**b**) A representative image of Western blot to detect purified Nb4. Purified Nb4 was loaded on the SDS-PAGE gel and then transferred to a PVDF membrane. The membrane was probed with the primary antibody anti-His-FITC (Beijing CW Biotechnology Ltd. Co.).

Human lung tissues expressed abundant SP-A protein, which appeared as protein bands with molecular weights of 35 kDa and 70 kDa on the SDS-PAGE gel (Fig. 1a). ELISA further confirmed the abundant SP-A expression in human lung tissues (Fig. 1b). We used human lung tissues to screen our previously constructed Nb library and enrich Nbs that can bind human lung tissues. Three rounds of biopanning resulted in an Nb sub-library of 3.8 × 10^6^ CFU (Table S1). We then further screened this Nb sub-library using a phage-ELISA assay, which included human pulmonary tissue total protein extracts as the antigen source and mouse anti-human SP-A (SP-A-mAb) as the positive control antibody, and identified 15 positive monoclonal Nbs (Fig. 2a). Peptide sequencing analysis revealed 4 IgG2a subtype clones and 11 IgG3 subtype clones (Fig. 2b). Cluster analysis of the 15 peptide sequences found 7 different sequences and 2 overlapping sequences (Nb4 and Nb28) (Fig. 2c). Thus, we focused on Nb4 in the rest of our study and used recombinant expression vector to expand Nb4. Nb4 was highly soluble and had a molecular weight of approximately 19 kDa (Fig. 3a and b).

### Nb4 was as specific as commercial available mouse anti-human SP-A (SP-A-mAb) in *in vitro* assay

**Figure 4:**
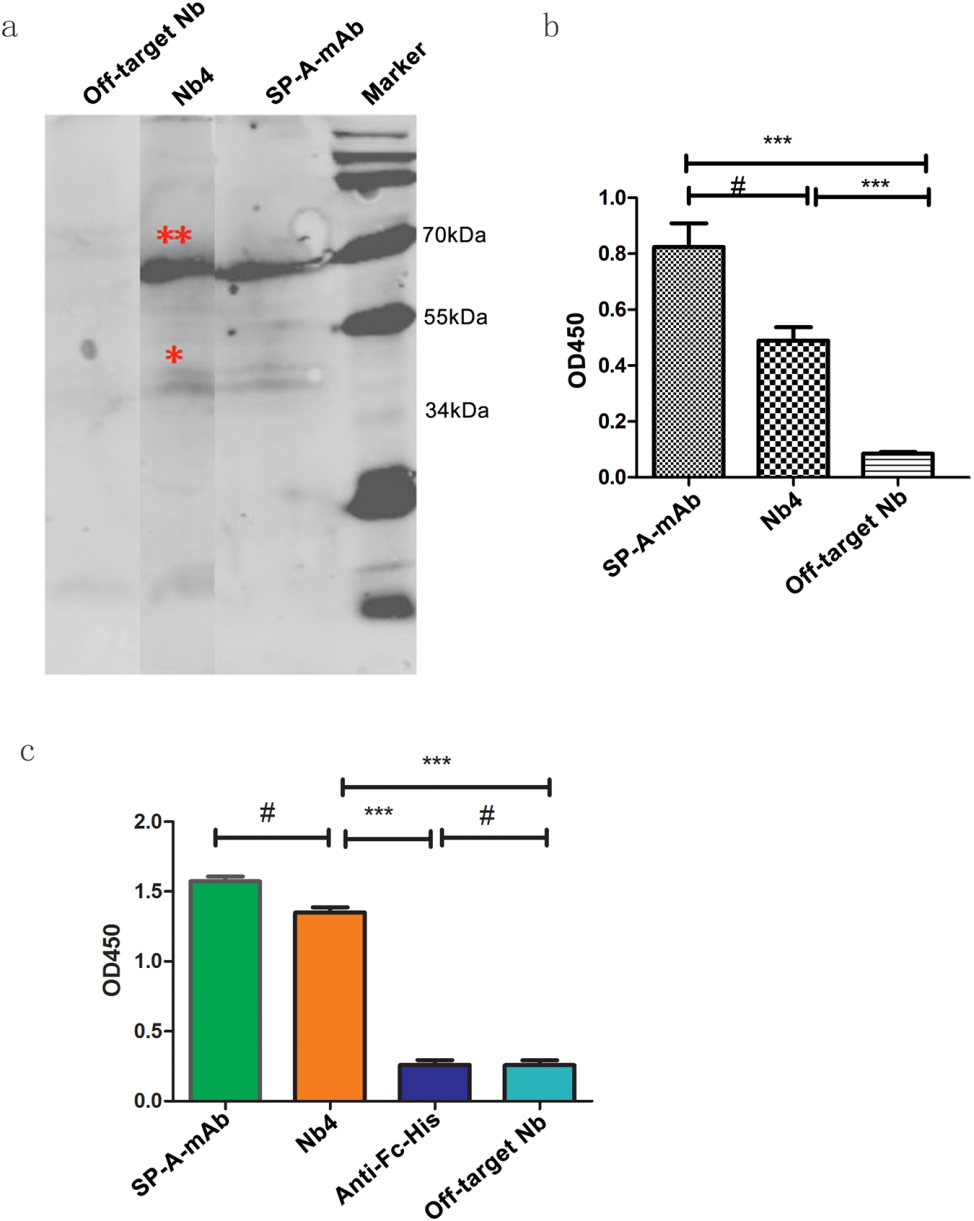
Nb4 specifically reacted with human SP-A. (**a**) Representative image of Western blot showing that Nb4 specifically bound to human lung SP-A. Human pulmonary tissue total protein extracts were loaded on SDS-PAGE gel and transferred to PVDF membranes. The membranes were probed with SP-A-mAb, Nb4, and off-target Nb. (**b**) ELISA assay on human lung total protein extracts. Human lung tissue total protein extracts were added in 96-well plates. The plates were incubated with primary antibodies SP-A-mAb, Nb4, and off-target Nb and the corresponding secondary antibodies. (**c**) ELISA assay on recombinant human SP-A protein. Recombinant human SP-A protein was prepared and then was added to 96-well plates. The plates were then incubated with SP-A-mAb, Nb4, anti-Fc-His, and off-target Nb. ***P < 0.05, ^#^P > 0.05.

**Figure 5:**
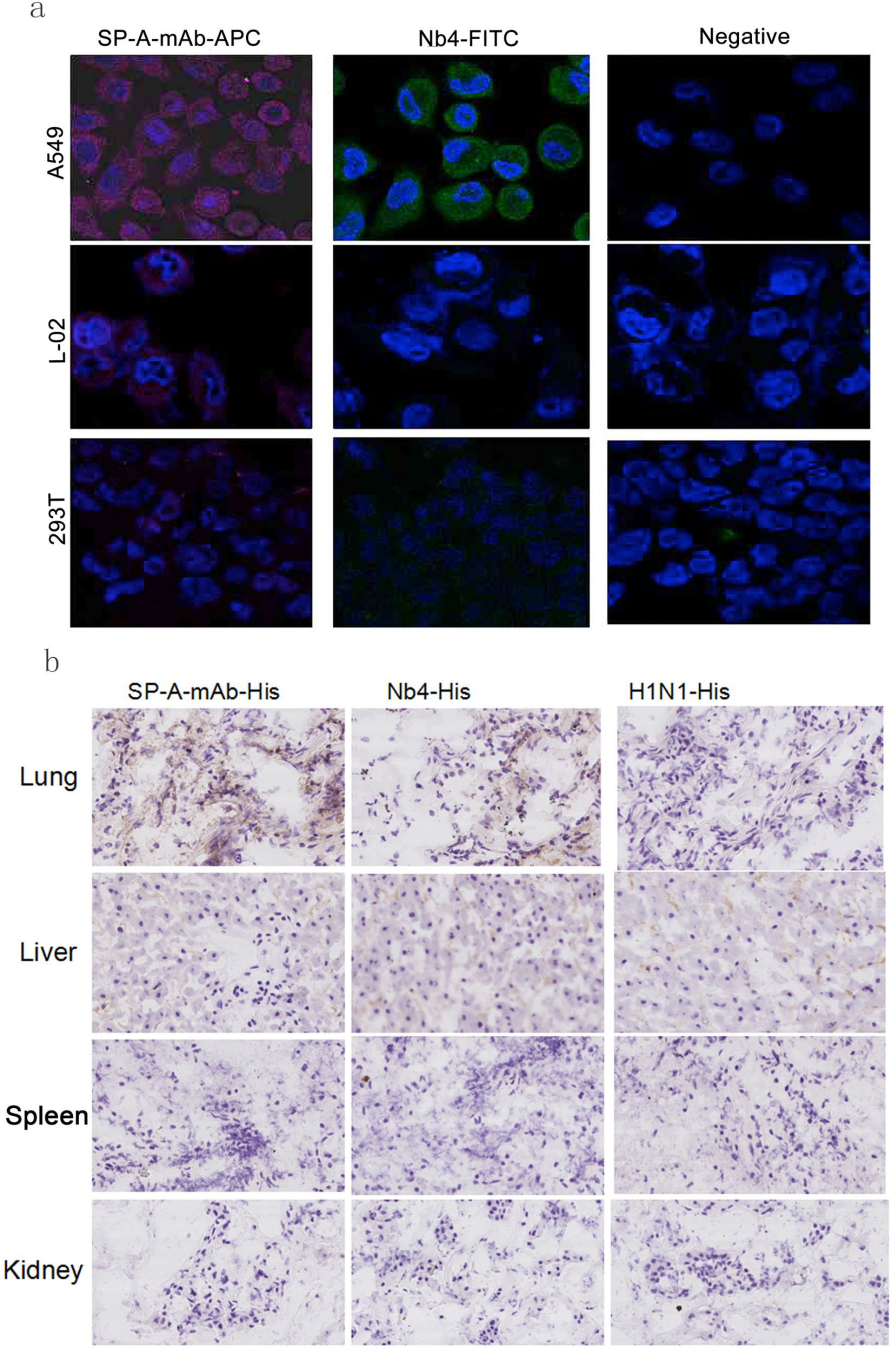
Immunofluorescent staining and immunehistochemical staining with Nb4. (**a**) Human epithelial A549 cells were positively stained with Nb4. Human lung epithelial A549 cells, human liver L-02 cells, and human kidney 293 T cells were fixed and stained with SP-A-mAb, Nb4-FITC, and off-target Nb (negative). (**b**) Human lung tissues were positively stained with Nb4. Human lung, liver, spleen, and kidney tissue specimens were stained with SP-A-mAb, Nb4-His, and H1N1-His (off-target control).

Western blot showed that Nb4 and SP-A-mAb specifically reacted with the SP-A protein in human lung tissue homogenates, which appeared as proteins bands with molecular weights of 35 kDa and 70 kDa on the SDS-PAGE gel (Fig. 4a). ELISA assay on human lung tissue protein extracts further confirmed the specific binding of Nb4 to human SP-A protein (Fig. 4b). We then prepared recombinant human SP-A proteins. The human SP-A gene was used to construct a recombinant vector expressing human SP-A (PET32-SPA). Bacteria were transformed with the recombinant expression vector, and the recombinant human SP-A protein was purified. The purified recombinant human SP-A protein showed a molecular weight of approximate 41 kDa (Supplementary Fig. S1) on the SDS-PAGE gel and interacted with the commercial SP-A-mAb specifically (Supplementary Fig. S2). We then used the recombinant human SP-A protein as antigen for ELISA assay. Both Nb4 and SP-A-mAb bound the purified recombinant human SP-A protein efficiently (Fig. 4c). Furthermore, we have used the ForteBio’s Octet System to investigate the binding affinity of Nb4 to the purified recombinant SP-A. We found that the Kds of Nb4 and SP-A-mAb were 7.48 × 10^−7^ M and 1.38 × 10^−9^ M, respectively, suggesting that the affinity of Nb4 to the recombinant SP-A protein appears to be lower than SP-A-mAb. These data support that Nb4 can bind human lung tissues and recombinant human SP-A. Off-target Nb did not react with human lung tissue homogenates, total protein extracts, and purified human lung SP-A protein (Fig. 4). To further evaluate the specificity of Nb4 to the lung, we performed immunocytochemical staining on human lung epithelial A549 cells, human liver L-02 cells, and human kidney 293 T cells. A549 cells were positive for Nb4-FITC (green) and SP-AmAb-APC (red), whereas L-02 and 293 T cells were negative for Nb4 and SP-A-mAb (Fig. 5a). The off-target Nb did not react with the three cell lines (Fig. 5a). IHC of human lung, liver, spleen, and kidney tissue specimens demonstrated that only lung tissue specimen showed strong positive staining for Nb4 and SP-A-mAb, whereas tissue specimens of the other organs did not react with Nb4 and SP-A-mAb (Fig. 5b).

### Nb4 accumulation in nude mice and toxicity in rats

**Figure 6:**
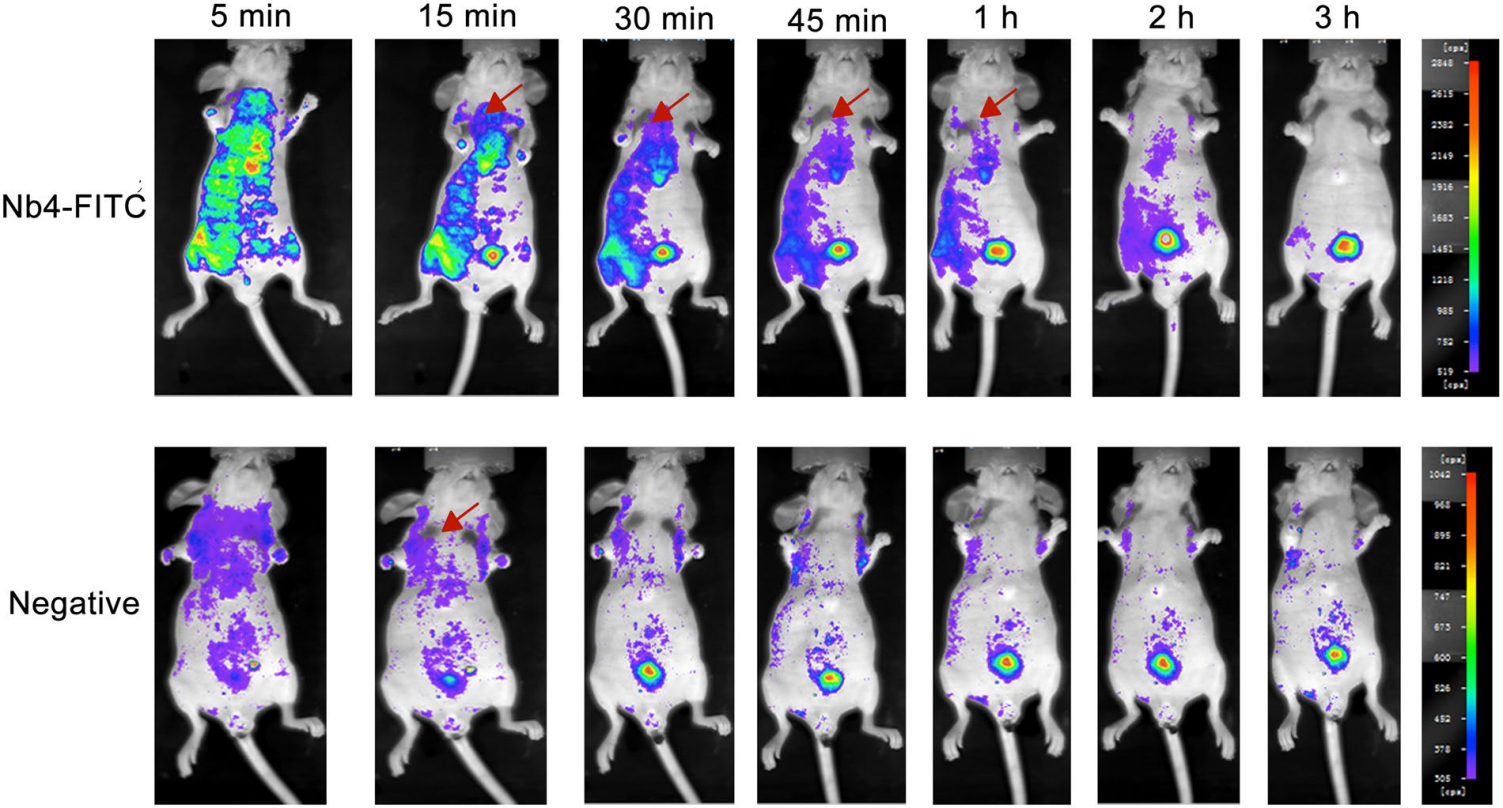
Distribution of Nb4 in nude mice. Nb4-FITC (10 μL, 1 mg/kg) was injected into nude mice via the tail vein. Mice in control group were injected with an equal amount of H1N1-FITC. The distribution of Nb4-FITC and H1N1-FITC was observed at 5 min, 15 min, 30 min, 45 min, 1 h, 2 h, and 3 h after the injection under a Xenogen IVIS Imaging System (PerkinElmer, MA, USA). Red arrows are pointing to the lung.

**Figure 7:**
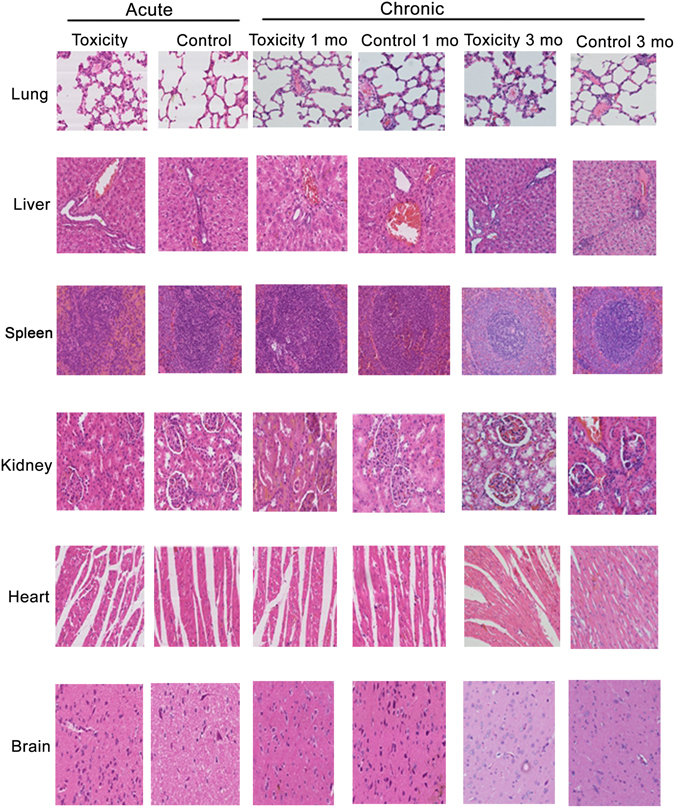
Nb4 showed low toxicity in rats. For the evaluation of acute Nb4 toxicity, rats (4-week old) were injected with 10 mg/kg Nb4 (10-fold higher than the normal dose) via the tail vein, or injected with equal volume of saline. One week after the injection, liver and kidney functions and the pathologic changes in the heart, liver, spleen, lung, kidney, and brain were examined. For the assessment of chronic Nb4 toxicity, rats were injected with Nb4 (1 mg/kg) via the tail vein once daily for one month or 3 months. Rats in the control group received an equal dose of normal saline. Liver and kidney functions and the pathologic changes in the heart, liver, spleen, lung, kidney, and brain were examined.

Because human and mouse SP-A share very high homology, we tested Nb4 distribution in nude mice. A total of 200 μL Nb4-FITC (1 mg/kg) was injected into nude mice via the tail vein. Nb-H1N1-FITC was used as an off-target negative control. Nb4-FITC accumulated at the highest extent in the lung 15 minutes after the injection, and the accumulation lasted 2 hours. Nb4-FITC was excreted 3 hours after the injection. Because the negative control Nb is not specific, it was excreted faster than Nb4. Thus, the signals of Nb4-FITC inside mice were stronger than those of the negative control Nb after injection. Since SP-A is also expressed in nude mouse kidney, Nb4-FITC accumulated in mouse kidney as well. Nb4 is excreted through urine, thus resulting in Nb4 accumulation in mouse bladder. In contrast, the off-target control Nb did not accumulate in any specific organ (Fig. 6). Toxicity test showed that serum levels of alanine aminotransferase, aspartate transaminase, and serum creatinine were increased significantly 3 months after an injection of 1 mg/kg Nb4 once daily (*P* < 0.05, Table 1), suggesting that Nb4 may cause liver and renal toxicity for long-term use. Histopathological examination found only mild pulmonary interstitial inflammation one month and three months after the injection compared with the control (Fig. 7).

**Table 1:** Serum ALT, AST, BUN and Cr levels in rats.

Group	AST	ALT	Cr	BUN
Acute Nb4	119 ± 13.91	43.75 ± 7.15	36 ± 2.92	8.83 ± 0.56
Acute Control	137 ± 27.34	45.5 ± 4.39	30.25 ± 8.53	9.33 ± 0.80
Chronic Nb4 one month	141.25 ± 13.44	41.2 ± 6.49	24.4 ± 2.06	9.8 ± 2.50
Chronic control one month	129 ± 31.29	49.6 ± 8.45	19 ± 1.41	8.08 ± 1.18
Chronic Nb4 3 months	228.67 ± 40.11*	60.33 ± 13.96	32.83 ± 4.30*	6.68 ± 0.69
Chronic control 3 months	163.83 ± 21.78	70.33 ± 39.48	25.17 ± 1.34	7.0 ± 0.78

*P < 0.05 vs. the control. AST: aspartate aminotransferase; ALT: Alanine transaminase; Cr: Creatinine; BUN: blood urea nitrogen.

### Physical and chemical characteristics of Nb4

**Figure 8:**
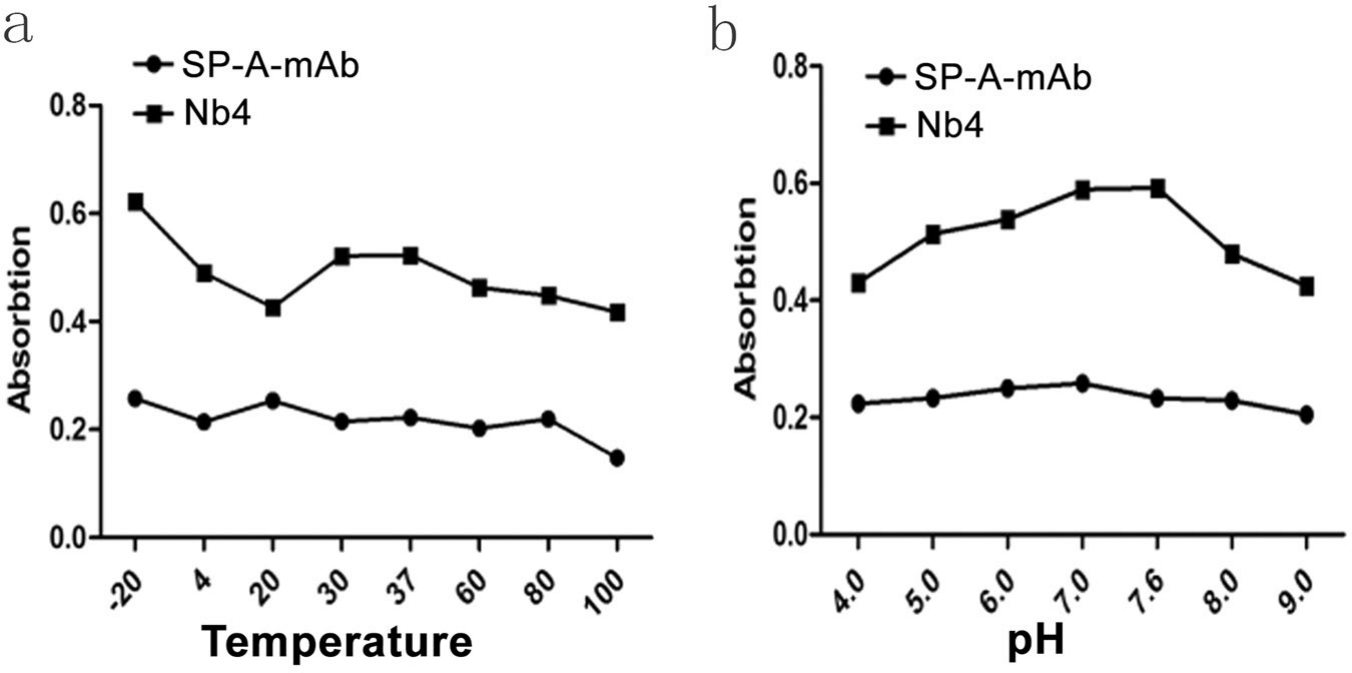
Physical properties of Nb4. (**a**) The effects of temperature on Nb4 stability. (**b**) The effects of pH on Nb4 stability. Nb4 was treated at different pH and temperature, and then was used for ELISA assay with human lung tissue total protein extracts as the antigen source.

The size of Nb4 particle was 10 nm × 9.5 nm × 9 nm based on TEM (Supplementary Figure S3). The maximum diameter of Nb4 dimer was 20.5 nm (Supplementary Figure S3). The Nb4 appeared more sensitive to temperature and pH than SP-A-mAb (Fig. 8a and b). The binding activity of Nb4 reduced sharply when temperature increased from −20 °C to 20 °C, and maintained at a relatively stable level between 30 °C–37 °C (Fig. 8a). The binding activity of Nb4 increased when pH increased from 4.0 to 7.0 and was stable between pH 7.0 and pH 7.6 (Fig. 8b).

## Discussion

In the current study, we identified Nb4, which can specifically target human lung tissues by binding to the antigen SP-A protein. SP-A, an alveolar surfactant protein secreted by alveolar type II epithelial cells and Clara cells, is abundantly expressed in human lung tissues, accounting for 50% of total alveolar surfactant protein^16,17^. Because of its high abundance and specificity to the lung, SP-A is often considered as an ideal pulmonary targeting ligand for drug delivery^18–21^. In our previous study, we successfully constructed an Nb library for rat pulmonary surfactant protein A and identified Nb17 as a specific Nb for rat pulmonary SP-A. Nb17 is very specific, shows high affinity to the target protein, and has a satisfactory safety profile^22^. The current study was based on our previous experience on Nb17 and took the advantages of the very high homology (98%) of rat and human pulmonary SP-A protein to identify Nb4, which specifically binds human pulmonary SP-A.

Nb4 in the current study had a molecular weight of 19 kDa and bound human pulmonary cell lines and tissues very specifically. The target-binding specificity of Nb4 was comparable to the commercial available SP-A-mAb. Short-term exposure to Nb4 caused no obvious renal and liver toxicity in rats. In addition, the lung-specific accumulation of Nb4 was further confirmed in nude mice. We found that Nb4 rapidly accumulated in mouse lung 5 minutes after the injection of Nb4 and then was excreted through the kidneys and urinary bladder gradually within 3 hours after the injection. No apparent Nb4 retention was found in other organs, such as liver and spleen, further supporting the lung-specific targeting of Nb4. Thus, Nb4 may be used as a vector to specifically deliver drugs to the lung.

Pulmonary Nb drug delivery system has been reported previously. For example, ALX-0171 has been developed to treat respiratory syncytial virus (RSV) infections^23^. Detalle *et al*. have found that ALX-0171 inhibited RSV proliferation by 87% whereas only 18% of the virus proliferation can by suppressed by the monoclonal antibody palivizumab^24^. Nb drug delivery technology has also been tested to treat breast cancer. Tam-loaded PVP–PLGA NPs and herceptin-conjugated Tam-loaded PVP–PLGA NPs, which target HER2, have been found to treat multi-drug-resistant breast cancer, and the Nb targeting F-actin capping protein CapG inhibits cancer metastasis in animal models of breast cancer^25,26^. In addition, the EGFR-targeting Nbs, 17864-L(x)-EGa1-PEG and AG538-loaded nanobody-liposomes, have also been tested as anti-cancer agents^27–29^.

Compared to conventional SP-A antibody, Nb4 has great advantages. Conventional antibodies, including polyclonal and monoclonal antibodies, usually have a high molecular weight (150 kDa), resulting in poor tissue penetration when they are used as drug delivery vectors. In addition, conventional antibodies also have strong immunogenicity. These limitations considerably dampen the enthusiasm of using antibodies as drug delivery vectors although the specific antigen-antibody binding is a very efficient targeted delivery mechanism. Nb may be a promising alternative to overcome those limitations associated with antibody vectors while still maintain the advantage of specific antigen-antibody binding. In addition to enhance the drug concentration in the target tissues, Nb appears to be safe and have low toxicity based on animal model studies. Ulrichts *et al*. have demonstrated that the Nb ALX-0081 completely inhibited the adhesion of human platelets that were isolated from patients with acute coronary syndrome^30^. In a baboon model of acute thrombosis and surgical bleeding, ALX-0081 appears to show good efficacy and have a satisfactory safety profile^30^. In a preclinical baboon model of acquired thrombotic thrombocytopenic purpura, Callewaert *et al*. found that anti-VWF Nb ALX-0681 successfully treated the disease without evident risk of severe bleeding^31^. Although Nbs have advantages, clinical application of Nbs remains to be determined.

## Conclusion

The current study first identified Nb4, which was a stable 19 kDa nanobody that specifically targeted human lung tissues by binding the antigen SP-A. In addition, Nb4 also specifically accumulated in the lung of nude mice and cause no renal and liver toxicity in rats at short-term exposure. These findings suggest that Nb4 may be a promising vector for lung-targeting drug delivery.

## Materials and Methods

### Animals

The protocol for animal experiment and maintenance has been approved by the Institutional Animal Care and Use Committee of Shanghai Pulmonary Hospital of Tongji University School of Medicine (Approval No: SYXK [SH] 2012-0031). A total of 4 female BALB/c-nu/nu mice (5-week old, 18–20 g) and 27 female rats (4–5-week old, body weight 110 ± 15 g) were provided by Shanghai SLAC Laboratory Animal Co., Ltd. (Experimental animal production license number: SCXK [SH], Shanghai, PRC).

### Human tissue acquisition

The protocol for human tissue acquisition and handling has been approved by the Institutional Review Board of Shanghai Pulmonary Hospital of Tongji University School of Medicine (Approval No: 2011-FK-10). Written informed consent was obtained from all the patients. Human lung tissue specimens were from Shanghai Pulmonary Hospital. Human heart, kidney, liver, spleen, and brain tissue specimens were from the Tenth People’s Hospital of Tongji University. Immunohistochemical (IHC) staining was performed by Google Biotechnology Co., Ltd.

### Reagents

Bacterial strains *E*. *coli* TG1, *E. coli* DH5α, and *E. coli* BL21 were purchased from Amersham Biosciences Company (NJ, USA). The vectors pET-44a and pET-26b (+) were purchased from Tiangen Biotech (Beijing, China) and Novegen company (Germany), respectively. PCR premix taq, DNA markers, IPTG, and DNA ligation kit were obtained from TaKaRa Bio Inc. (Dalian, Liaoning, PRC). The restriction endonucleases (BamH1 and XhoI) were manufactured by Shanghai Yitao Biological Instrument Co. Ltd. (Shanghai, PRC). M13K07 helper phage was obtained from Beijing Biocoen Biotechnology Co, Ltd. (Beijing, PRC). The kits for plasmid extraction, gel extraction, and PCR purification were from Shanghai Haojia Science & Technology Development Co., Ltd (Shanghai, China). The anti-human SP-A monoclonal antibody (SP-A-mAb, 6F10), anti-His-FITC antibody, anti-mouse IgG-FITC antibody, and 4′,6-diamidino-2-phenylindole (DAPI) were from Abcam Company (Cambridge, UK). Goat anti-mouse lgG-HRP, anti-His tag mouse monoclonal antibodies, and HRP-labeled anti-His tag mouse monoclonal antibodies were purchased from Jiangsu Kangwei Biotech Co., Ltd. (Taizhou, Jiangsu, China). Goat anti-mouse IgG conjugated with IR Dye 800CW was bought from LI-COR Biosciences (Lincoln, NE, USA). Normal goat serum, tissue lysis buffer, bovine serum albumin standard, kanamycin sulfate powder, and ampicillin powder were from Tiandz, Inc. (Beijing, China). TMB substrate solution was from R&D Systems, Inc. (Minneapolis, MN, USA). Nitrocellulose membrane was from Shanghai Minipore Industrial Co., Ltd. (Shanghai, China).

All experimental procedures were carried out in accordance with the guidelines of the Ethic Committee of Shanghai Pulmonary Hospital (Approval number 2014FK04, Approval date:February 25th, 2014).

### Preliminary biopanning of a previously constructed phage-display nanobody (Nb) library to enrich Nbs binding to human pulmonary tissues

Human pulmonary tissue frozen sections were used for biopanning of our previously constructed phage-display Nb library^22^. The frozen tissue sections were washed 10 times with sterile PBS and then incubated with 2 mL solution containing the Nb library at 4 °C overnight. Next day, the tissue sections were washed with PBS buffer for 10 times, and then the attached phages on the tissue sections were eluted with 5 mL 2-Yeast-Tryptone-Ampicillin-Glucose (2YTAG). The elution was diluted in 100 mL 2YTAG and cultured in a shaker at 37 °C until OD_600_ of the culture = 0.6. The culture was then centrifuged at 4,500 rpm for 5 minutes. The supernatant was discarded. The pellets were re-suspended in 12 mL media (the titer should be approximately 10^13^), incubated at 37 °C for 30 minutes, and diluted with 200 mL 2-Yeast-Tryptone-Ampicillin-Kanamycin (2YTAK). The suspension was incubated in a shaker at 37 °C until OD_600_ = 0.8, and then centrifuged at 4,500 rpm at 4 °C for 15 minutes. The supernatant was collected, mixed with 1/5 volume of PEG800 suspension, and kept on ice for one hour. The mixture was centrifuged at 9,000 rpm at 4 °C for 30 minutes. The pellets were collected, re-suspended in 15 mL 2-Yeast-Tryptone (2YT), and centrifuged at 3,000 rpm at 4 °C for 5 minutes. The supernatant was collected as the first-round biopanning product. A total of 3 rounds of the biopanning were performed. The second and third round of biopanning contained 15 and 20 times of washing, respectively. The selected phages from the third round biopanning were used to infect TG1 competent bacterial cells. The infected TG1 bacteria were spread on agar plates, and monoclones were selected.

### Preparation of human pulmonary tissue total protein extracts (SP-A antigen)

Two grams of human pulmonary tissue were cut and suspended in 10 mL RIPA buffer (50 mM Tris,150 mM NaCl, 1% Triton X-100, 1% sodium deoxycholate, 0.1% SDS, sodium orthovanadate, sodium fluoride, EDTA, leupeptin, Phenylmethanesulfonyl fluoride, pH 7.4). The mixture was then aliquoted into 10 1.5-mL eppendorf tubes. Each aliquot was homogenized using a sonicator (60 Hz, 90 s/cycle for 2 cycles) and then centrifuged at 12,000 rpm for 5 min at 4 °C. Total protein concentration in the supernatant was determined using the bicinchoninic acid assay (BCA assay). The expression of human pulmonary SP-A was determined by Western blot and ELISA.

### Binding of Nb4 to recombinant human SP-A proteins

Recombinant human SP-A protein was constructed. The human SP-A gene, which was provided by Shanghai YouLong Biotech (Shanghai, China), was used to construct a recombinant expression vector PET32-SPA (BL21). Bacteria were transformed with the recombinant expression vector, and the recombinant human SP-A protein was purified and analyzed on SDS-PAGE gel and in ELISA assay. In the ELISA assay, a 96-well plate was coated with the purified recombinant SP-A proteins (200 µg/mL per well) at 4 °C overnight. The plate was then washed with PBS buffer for 5 times (3 minutes per time) and blocked with 2% BSA in PBS at 37 °C for 2 hours. Nb4-His, SP-A-mAb, anti-Fc-His, and off-target control Nb (300 µL) were added to the plate. The plate was incubated at 37 °C for 2 hours. After washing, the secondary antibody anti-mouse his-HRP was added to the blank wells and wells treated with Nb4-His or anti-Fc-His, and the secondary antibody anti-mouse IgG-HRP was added to the wells treated with SP-A-mAb. The plate was incubated at 37 °C for 45 minutes and washed with PBS. Color was developed with TMB. The plate was read at 450 nm in a plate reader. ForteBio’s Octet System (Cold Spring Biotech Corp, USA) was used to analyze the binding affinity of Nb4 and SP-A-mAb to the purified recombinant human SP-A. Kds were determined.

### Biopanning and expansion of anti-human pulmonary SP-A Nb

The human pulmonary tissue total protein extracts (SP-A antigen) were diluted (1: 2) with carbonate buffer (pH 9.6) and used to coat 96-well plates (200 µL per well). Mouse anti-human SP-A antibody (SP-A-mAb) was used as the positive control antibody, and off-target Nb (Nanchang Da Jia Biotech Ltd. Co.) was used as the negative control. The plates were then incubated at 4 °C overnight. The plates were drained and blocked with 5% skim milk in PBS for 2 hours at 37 °C. After blocking, the plates were washed with PBS for 5 times. Phage-ELISA was performed to select the positive clones for human SP-A. M13K07 helper phages (containing Nbs), which were released from the bacterial monoclones collected from the third round preliminary biopanning, were added to the plates. The plates were incubated at 4 °C overnight and washed with PBST for 4 times. A total of 200 μL of HRP conjugated anti-M13K07 monoclonal antibodies (1:5000 dilution) was added to the plates. The plates were incubated at 37 °C for one hour and washed with PBST for 3 times. The color was developed by adding 200 μL TMB color developing buffer to each well and the plate was incubated at room temperature for 15 minutes. The plates were read in a plate reader at 450 nm. Wells with a value of OD450 > 3 times of the negative value were considered to contain a positive clone. The peptides of the positive clones were then sequenced. Two clones (Nb4 and Nb28) showed highly overlapping peptide sequences, and Nb4 was used for further analysis.

Nb4 was expanded by constructing recombinant expression pET-26b (+)-V_HH_ vectors containing the DNA sequences for the peptides in Nb4. Briefly, recombinant plasmids PMD19-T-V_HH_ containing the target fragments were first constructed and transformed into *E. coli* DH5α. The target fragments (∼500 bp) were isolated by endonuclease digestion of the recombinant plasmids and inserted into pET-26b (+). The recombinant pET-26b (+)-V_HH_ was subsequently transformed into *E. coli* BL21 to overexpress the target peptides. Total proteins from the bacterial culture were extracted, analyzed by SDS-PAGE, Western blot, and ELISA.

### Western Blot

Human frozen pulmonary tissue were homogenized, and total proteins of the tissue homogenates were separated by SDS-PAGE gel and transferred to a PVDF membrane. After blocking, the membrane was incubated with the following primary antibodies: Nb4, mouse anti-human SP-A monoclonal antibody (SP-A-mAb), and anti-H1N1-Nb (off-target Nb) overnight at 4 °C. The membrane was then washed with PBST and incubated with the secondary antibodies, HRP-conjugated anti-mouse IgG and anti-His-HRP for 2 hours. After washing, target protein signals on membrane were visualized by chemiluminescence method and analyzed by the Bio-Rad imaging system.

### ELISA

Ninety-six-well plates were coated with 2 mg/mL of human pulmonary tissue total protein extracts at 4 °C overnight and incubated with Nb4, SP-A-mAb, and anti-H1N1-Nb at 37 °C for 2 h. After washing with PBS, the plates were incubated with the secondary antibodies anti-mouse IgG-HRP and anti-His-HRP for 45 minutes. Then, the plates were washed with PBS. TMB substrate solution was added to develop color and sulfuric acid was added to terminate the reaction. The absorbance at 450 nm (OD450) was determined in a microplate reader.

### Immunocytochemical staining

Human pulmonary epithelial A549 cells, liver L-02 cells, and kidney 293T cells were used for the staining. Cells at 95–100% confluence were fixed and incubated with the primary antibodies, SP-A-mAb, Nb4-FITC, and anti-H1N1 Nb-FITC. After washing, the cells were incubated with the secondary antibodies anti-mouse IgG-APC. Nuclei were stained with DAPI. The stained cells were observed under a Nikon LSCM system (Nikon C2 plus LSCM, Tokyo, Japan).

### Immunohistochemical staining (IHC)

Frozen tissue sections of human lungs, livers, spleens, and kidneys were fixed and incubated with the primary antibodies SP-A-mAb (positive control), Nb4, and anti-H1N1 Nb (off-target negative control). Goat anti-mouse IgG-HPR and mouse anti-His-HRP monoclonal antibodies were used as the secondary antibodies. The stained sections were observed under a Leica section scanner (Leica SCN 400, Leica Microsystems Ltd, Germany).

### Nb4 distribution in nude mice

Nb4 was labeled with FITC (Nb4-FITC). The distribution of Nb4-FITC in nude mice was determined. Mice were anesthetized by inhalation of isoflurane. Ten μL Nb4-FITC (1 mg/kg) was injected via the tail vein. Mice in control group were injected with an equal amount of H1N1-FITC. The distribution of Nb4-FITC and H1N1-FITC was observed at 5 min, 15 min, 30 min, 45 min, 1 h, 2 h, and 3 h after the injection under a Xenogen IVIS Imaging System (PerkinElmer, MA, USA).

### Acute Nb4 toxicity test

Rats (4-week old) were injected with 10 mg/kg Nb4 (10-fold higher than the normal dose) via the tail vein, or injected with equal volume of saline. Six rats were used in each group. One week after the injection, liver and kidney functions and the pathologic changes in the heart, liver, spleen, lung, kidney, and brain were examined.

### Chronic Nb4 toxicity test

Rats were randomized into one-month test group, three-month test groups, and control group. The rats in the chronic toxicity test groups were injected with Nb4 (1 mg/kg) via the tail vein once daily for one month or 3 months, and rats in the control group received an equal dose of normal saline. Liver and kidney functions and the pathologic changes in the heart, liver, spleen, lung, kidney, and brain were examined.

### Physical and chemical characteristics of Nb4

The particle size of Nb4 was measured. Nb4 solution was filtered through a molecular sieve to remove protein contaminants and desalted. Then, Nb4 was labeled with gold, dried, and counter-stained with phosphotungstic acid. The size, shape, and homogeneity of Nb4 were determined by transmission electron microscopy (TEM). The thermal stability and acid-base resistance of Nb4 was also determined. SP-A-mAb and Nb4 (0.1 mg/mL) were maintained at −20 °C, 4 °C, 20 °C, 30 °C, 37 °C, 60 °C, 80 °C, or 100 °C to test thermal stability, and were placed in buffers at pH 4.0, 5.0, 6.0, 7.0, 7.6, 8.0, or 9.0 for 1 h. The absorbance at 450 nm were determined by ELISA.

### Statistical analyses

All experimental data were analyzed by SPSS 16.0. Continuous variables are presented as mean ± standard deviation (SD). One-way analysis of variance was performed for multiple-group comparisons. Least significant difference t-test was used for further analysis when significant differences existed among different groups. *P* < 0.05 was considered statistically significant.

## Electronic supplementary material

supplementary information
