# Erratum: Identification of a nanobody specific to a human pulmonary surfactant protein A

**DOI:** 10.1038/s41598-017-12271-0

**Published:** 2017-09-27

**Authors:** Xian He, Shan-Mei Wang, Zhao Fang Yin, Meng-Meng Zhao, Nan Li, Feng Yu, Liu-Sheng Wang, Yang Hu, Yu-Kui Du, Shan-Shan Du, Yan Li, Ya-Ru Wei, Shan-Shan Chen, Jian-Hua He, Dong Weng, Hui-Ping Li

**Affiliations:** 1 Department of Respiratory Medicine Suzhou University, School of Medicine, SuZhou, China; 2 Department of Respiratory Medicine, Shanghai Pulmonary Hospital Tongji University, School of Medicine, Shanghai, China; 3 Shanghai Institute of Applied Physics, Chinese Academy of Sciences, Shanghai, China; 4 Present Address: Department of Respiratory Medicine The Sixth People’s Hospital of Nantong, Suzhou University, School of Medicine, SuZhou, China

*Scientific Reports* **7**:1412; doi:10.1038/s41598-017-01456-2; Article published online 03 May 2017

The original PDF version of this Article contained an error in the order of corresponding authors:

“Correspondence and requests for materials should be addressed to D.W. (email: cruise00@126.com) or H.-P.L. (email: liw2013@126.com)”

now reads:

“Correspondence and requests for materials should be addressed to H.-P.L. (email: liw2013@126.com) or D.W. (email: cruise00@126.com)”.

This has been corrected in the PDF version of this Article; the HTML version was correct at the time of publication.

