# Supplementary material for: Identification of a nanobody specific to human pulmonary surfactant protein A
Source: Sci Rep. 2017 May 3;7:1412. doi: 10.1038/s41598-017-01456-2 (PMC5431231; doi:10.1038/s41598-017-01456-2)
Supplement: Supplementary file 1 — supplementary information [file 41598_2017_1456_MOESM1_ESM.doc]

**Identification of a nanobody specific to human pulmonary surfactant protein A**

Xian He^1†#^, Shan-Mei Wang^2#^, Zhao Fang Yin^1#^, Meng-Meng Zhao^2#^, Nan Li^2^, Feng Yu^3^, Liu-Sheng Wang^2^, Yang Hu^2^, Yu-Kui Du^2^, Shan-Shan Du^2^, Yan Li^2^, Ya-Ru Wei^2^, Shan-Shan Chen^1^, Jian-Hua He^3^, Dong Weng^2&^, and Hui-Ping Li^2*^

**Affiliations**:

^1^Department of Respiratory Medicine

Suzhou University, School of Medicine, SuZhou, China

^2^Department of Respiratory Medicine, Shanghai Pulmonary Hospital

Tongji University, School of Medicine, Shanghai, China

^3^Shanghai Institute of Applied Physics, Chinese Academy of Sciences, Shanghai, China

†Present address:

Department of Respiratory Medicine

The Sixth People’s Hospital of Nantong, Suzhou University, School of Medicine, SuZhou, China

**# These authors contributed to this work equally**

***Corresponding author**:

Hui-Ping Li, MD, PhD

507 Zheng Min Road, Shanghai, 200433, China

Department of Respiratory Medicine, Shanghai Pulmonary Hospital

Tongji University, School of Medicine, Shanghai, China

Fax: 86-21-65111298

Phone: 86-21-65115006-2103

Email: liw2013@126.com

**^&^Co-corresponding author**:

Dong Weng, PhD

507 Zheng Min Road, Shanghai, 200433, China,

Department of Respiratory Medicine, Shanghai Pulmonary Hospital

Tongji University, School of Medicine, Shanghai, China

Fax: 86-21-65111298

Phone: 86-21-65115006-2206

**Supplementary Tables**

Table S1 Biopanning of the Nb library

| **Biopanning cycle** | **Number of sections** | **Phage input**  **(cfu)** | **Phage output (cfu)** | **Recovery rate (%)** |
| --- | --- | --- | --- | --- |
| 1 | 10 | 3.4 × 10^14^ | 1.2 × 10^9^ | 3.5 × 10^-6^ |
| 2 | 10 | 7 × 10^13^ | 7 .2 × 10^7^ | 9.7 × 10^-7^ |
| 3 | 10 | 5.2 × 10^13^ | 3.8 × 10^6^ | 7.3 × 10^-8^ |

Recovery rate = Phage output divided by phage input.

**Supplementary Figure S1 Image of a SDS-PAGE gel showing the purified recombinant human SP-A protein**


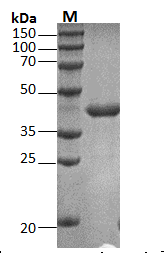


The recombinant human SP-A protein presented a molecular weight of approximately 41 kDa in the SDS-PAGE gel. M: molecular marker.

**Supplementary Figure S2 ELISA assay of the purified recombinant human SP-A protein and SP-A-mAb**


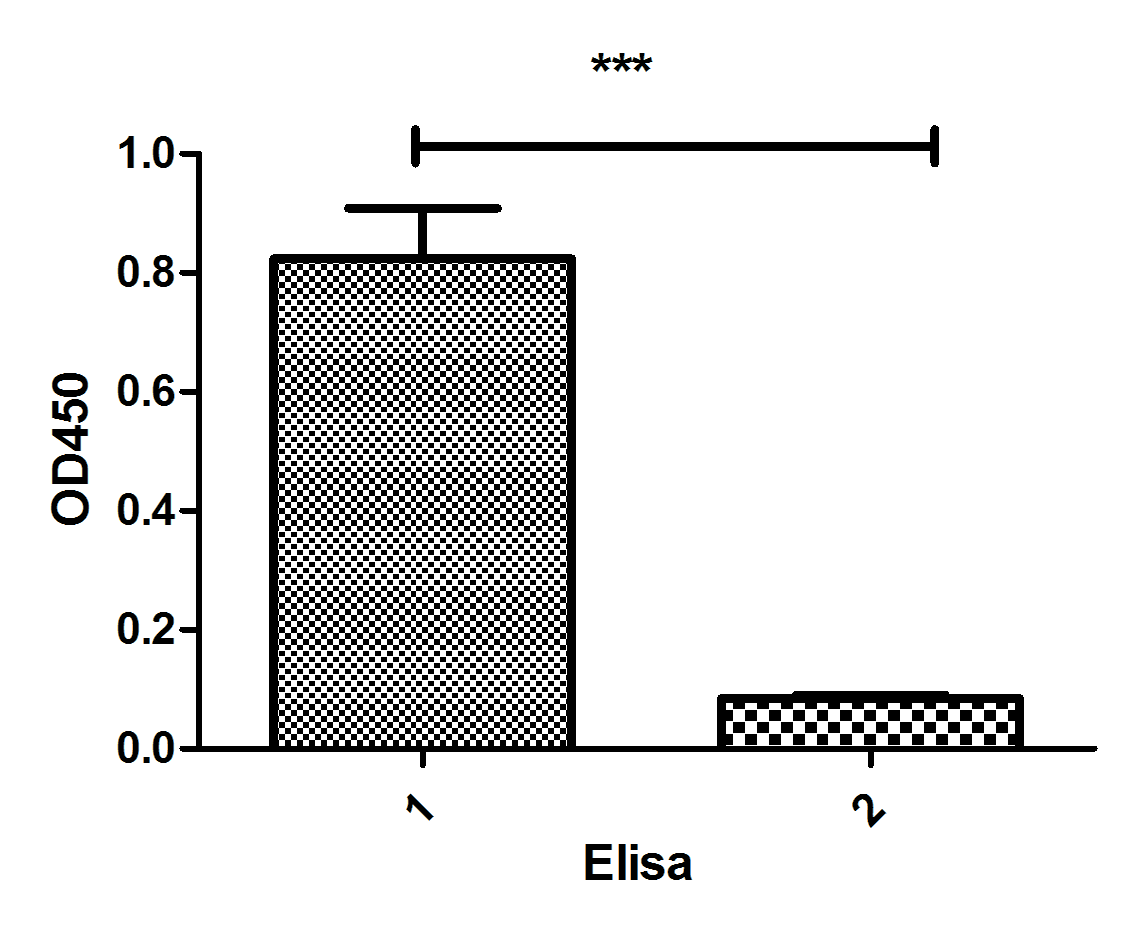


1: SP-A-mAb; 2: Control antibody. *** *P*<0.05.

**Supplementary Figure S3 A transmission electron microscopic image of Nb4 particle aggregates**

**

**

A representative transmission electron microscopic image showing that the dimension of Nb4 was 10 nm × 9.5 nm × 9 nm. The maximum diameter of Nb4 dimer was 20.5 nm.
